# Professional competency and moral courage among staff nurses

**DOI:** 10.1186/s12912-025-03197-4

**Published:** 2025-05-19

**Authors:** Eman Hassan Mohamed Ali, Marwa Abdelrahman Gaber Khalifa

**Affiliations:** Nursing Administration Department, Faculty of Nursing, Ain Shams University, Cairo, Egypt

**Keywords:** Professional competence, Moral courage, Staff nurses

## Abstract

**Background:**

Nurses must possess professional competency and moral courage to ensure the excellence of care and safety for patient, communicate effectively with other healthcare staff, and promote the establishment of consistent and universal care.

**Aim:**

This study aimed to investigate the relationship between professional competency and moral courage among staff nurses.

**Methods:**

A descriptive correlational study design was conducted at Ain Shams University Hospital, affiliated with Ain Shams University Hospitals. The study included 172 out of 310 staff nurses. Three tools were used for data collection, namely: Personal Characteristics Sheet, The Nurse Professional Competency Scale–Short Form (NPC-Scale SF), and The Nurses’ Moral Courage Scale (NMCS). Data were collected over a period of six weeks, starting from the second half of April 2024 until the end of May 2024.

**Results:**

This study revealed that 42.7% of the staff nurses were aged between 25 and 35 years, 61.4% were female, 28.7% held a nursing technical degree, and 51.5% had more than five years of experience. In addition, more than three-fifths of the participants reported a moderate level of professional competency, followed by a low level. Similarly, two-thirds of the participants reported a moderate level of moral courage, followed by a low level. Furthermore, there was a strong positive correlation between professional competency and moral courage among staff nurses (*r* = 0.637**, *p* = 0.000).

**Conclusion:**

This study concluded that there was a strong positive significant relationship between professional competency and moral courage among staff nurses, which answered the research question. This study recommends that training and educational programs should be conducted regularly and continuously to update and expand nurses’ knowledge and skills concerning professional competencies and the implementation of ideal models in clinical practice.

## Introduction

Recently, nursing has been identified as a complex field that demands a wide range of skills. Essentially, nurses are encouraged to continually enhance their professional competency [1]. The concept of competency is defined as a set of demonstrable characteristics and skills that enable and improve the efficiency of job performance [2]. Accordingly, professional competence is described as the ability of a person to handle specific scenarios or complete particular assignments or duties effectively. This ability encompasses competencies related to sensory‒motor abilities, mental processes, emotional aspects, character traits, and interpersonal abilities [3]. Moreover, nursing professional competency is characterized by a mix of abilities, understanding, orientations, principles, and skills that lead to efficient or superior outcomes in work and professional roles [4].

Importantly, professional competency involves providing nursing care that aligns with recognized professional standards. Achieving competency leads to improved quality of patient care and increased patient satisfaction with the nursing staff. In turn, this supports the advancement of nursing as a profession, the enhancement of nursing education, and the improvement of clinical practice. Moreover, higher levels of professional competency and satisfaction with care quality are associated with more favorable perceptions of the work environment, stronger adherence to ethical standards, greater career development potential, and increased commitment to the nursing profession [5].

Indeed, professional competency is seen as the accurate application of decision-making and routine actions involving knowledge, technical abilities, logical thinking in healthcare, interpersonal communication, emotional intelligence, ethical principles, and the ability to reassess daily practices to provide support to individuals and the broader community [6]. Competent nurses play a vital role in ensuring that healthcare services remain safe and efficient by integrating expertise, skills, and values that enable them to adapt to evolving healthcare environments. Therefore, nurses should primarily focus on the practical competencies applied during interactions with patients, healthcare professionals, and family members [7].

The dynamic and ethically complex field of nursing necessitates a careful balance between moral integrity and professional competence. Professional competency, which includes knowledge, skills, clinical judgment, and decision-making ability, is the cornerstone of high-quality patient care in the intricate and ever-evolving changing healthcare environment. Moral courage is defined as the ability to act ethically and advocate for patients’ rights and safety in the face of institutional pressure, fear, or adversity [8].

The American Association of Colleges of Nursing defines moral courage as the ability to uphold and apply ethical and moral principles when confronted with challenges. For nurses, moral courage is the determination to act on the basis of one’s ethical beliefs despite potential severe consequences. It is a crucial principle in nursing and a powerful strategy for addressing ethical dilemmas. Therefore, moral courage is closely associated with moral awareness, delivering safe, ethical, and effective patient care [9].

In the field of healthcare, nurses frequently encounter dilemmas involving conflicting ethical principles in their daily responsibilities [10]. Moreover, the growing recognition of patients, shifts in the healthcare requirements of communities, the pursuit of professional justice, and the availability of healthcare services have heightened the degree of ethical turmoil experienced by nurses, thereby necessitating significant moral courage in delivering secure and high-quality care while avoiding unethical practices [11]. Nonetheless, extensive research indicates that nurses are often burdened with unresolved ethical issues leading to moral turmoil, with severe instances even resulting in nurses abandoning their roles in nursing and quitting the profession altogether [12–13–14].

Moral courage also involves taking steps in line with ethical standards despite obstacles and risks in ethical situations, such maintaining patient confidentiality, delivering difficult news, or providing care for individuals with contagious illnesses. To effectively navigate and overcome such ethical challenges while fulfilling the moral obligations of the profession, nurses require moral courage. Those who possess moral courage intentionally and voluntarily make choices and take actions that benefit others, even when such actions may result in negative consequences for themselves or others [15].

Moral courage empowers healthcare providers to adhere to the ethical standards and values of their field in various instances, including protecting patient confidentiality, breaking distressing news, and managing care for patients with contagious diseases. For nurses to remain resilient in the face of both professional and ethical challenges, it is essential to assess their level of moral courage in order to gauge the strength of their core professional values [16]. In this context, moral courage is regarded as a key factor in delivering safe, systematic, and high-quality care, as it complements professional nursing competence by promoting high-quality patient care, ethical decision-making, effective advocacy, positive team collaboration, a healthy organizational culture, reduced burnout, and an enhanced institutional reputation [8].

Frontline caregivers play a crucial role in providing safe and professional care to clients. Nurses need professional competence with the aid of moral courage to assure the quality care and safety of patients, to interact with other healthcare professionals, and to advocate for consistent universal care with healthcare and community organizations [17]. Indeed, nurses encounter some moral problems in their daily work that need to be resolved to navigate the ethical and professional challenges inherent in their role. It becomes crucial to assess nurses’ moral courage, gauging the extent of their intrinsic professional values [18].

However, many studies have shown that nurses suffer from unresolved ethical problems that lead to moral distress with adverse consequences. In the most serious cases, this can result in nurses becoming disengaged from patient care or even leaving the profession entirely [5–9]. While moral courage has been recognized as a crucial component of nursing practice, it has received relatively limited attention, and surprisingly few studies have been conducted in this area [1–19].

The researchers noted that staff nurses had insufficient documentation of patients’ physical status and psychological status and were often capable of recognizing patients’ needs and relatives’ knowledge and experiences. In addition, low competency and a low level of communication with patients can affect moral courage. Therefore, this study was conducted to investigate nurses’ professional competence and moral courage.

### Aim of the study

This study aimed to investigate the relationship between professional competency and moral courage among staff nurses through:

- Assessing staff nurses’ self-reported professional competency level.
- Measuring staff nurses’ moral courage level.
- Finding out the relationship between professional competency and moral courage among staff nurses.

### Research questions

- What is staff nurses’ self-reported professional competency level?
- What is staff nurses’ moral courage level?
- What is a relationship between professional competency and moral courage among staff nurses?

## Methods

### Research design

A descriptive correlational design was used to achieve the aim of this study. Strobe checklist was used in this study.

### Research sample

The present study was conducted at Ain Shams University Hospital affiliated with Ain Shams University Hospitals in Cairo, Egypt. The hospital provides services across all medical specialties and consists of six floors, comprising 27 units /departments categorized into critical and non-critical units. The hospital has a total capacity of 618 beds. This study was conducted across all units and departments within Ain Shams University Hospital. The researchers employed a simple random sampling technique, selecting 172 out of 310 staff nurses using the following equation [20]:

$$\:n = \frac{{N \times \:P\left( {1 - p} \right)}}{{\left[ {N - 1\left( {{d_2}/{z_2} + p\left( {1 - p} \right)} \right)} \right]}}$$

n = sample size

N = population size

d = the error rate is 0.05

z = the standard score corresponding to the significance level is 0.95 and is equal to 1.96

p = availability of property and neutral = 0.50

### Inclusion criteria

Staff nurses worked full-time and had at least one year of experience in the previously mentioned settings were eligible to participate in the study. Only nurses working across all units and departments of the hospital were included.

### Exclusion criteria

Staff nurses with less than one year of experience were excluded. Additionally, nurses who declined to participate or did not provide informed consent were not included in the study.

### Data collection tools

#### Three tools were used for data collection

##### Tool 1: personal characteristics sheet of staff nurses

It was developed by researchers, age, sex, marital status, educational qualifications, years of experience, and attendance at training courses, were included.

##### Tool II: nurse professional competency scale-short form (NPC-Scale SF)

This scale was developed by Nilsson et al. [21]. and adapted by the researchers to assess staff nurses’ self-reported professional competency level. The validity and reliability of the tool were tested.

It is composed of 35 items divided into six sections. **Section 1**: Nursing care was included (5 items). (EX, autonomously apply the nursing process). **Section 2**: Values-based nursing care was included (5 items). (EX, respectfully communicate with patients, relatives and staff). **Section 3**: Medical and technical care were included (6 items). (EX, support patients during examinations and treatments). **Section 4**: Care pedagogic was included (5 items). (EX, provide patients and relatives with support to enhance sharing in patient care). **Section 5**: Documentation and administration of nursing care were included (8 items). (EX, make use of applicable data in patient records). **Section 6**: Leadership development and nursing care organization were included (6 items). (EX, Act sufficiently in the event of unprofessional conduct among employees).

Each item of subject response was measured on a 1–5 point Likert rating scale ranging from (1) indicating that it was extremely low, (2 low), (3 neutral), (4 high), and (5) indicating that it was extremely high.

For each area of scale, the scores of the items were summed, and the total score was divided by the number of items, resulting in a mean score for the part. These scores were converted into percent scores. The level was considered high if the percent score was more than 75%, moderate if the percent score ranged from 60 to 75%, and low if the percent score was less than 60% [21].

##### Tool III: the nurses’ moral courage scale (NMCS)

This scale was adapted by the researchers based on Numminen et al. [22]. This study aimed to measure staff nurses’ self-assessed moral courage level. The validity and reliability of the tool were tested.

It consists of 21 items, which are classified into four subscales as follows: 1-Compassion and true presence (5 items). (EX. I support a suffering patient by being truly present for him/her, even if it were to lead me to encounter my own inner fears). 2-Moral responsibility was (4 items). (EX. I participate in the care team’s ethical decision-making regardless of somebody else). 3- Moral integrity (7 items). (EX. I adhere to professional ethical principles even if I were to be bullied for it in my work unit). 4-Commitment to providing good care (5 items). (EX. If I observe evident deficiencies in someone else’s professional competence, I bring it up for discussion).

Each item of scale was measured on a five-point Likert scale: 1 = (Does not describe me at all); 2 = Describes me fairly little, 3 = Describe me on average, 4 = Describes me fairly well, and 5 (describes me very well). For each dimension, the total score was measured as follows: [< 60%] indicated a low level of moral courage, [≥ 60 – ˂75%] indicated a moderate level of moral courage, and [≥ 75%] indicated a high level of moral courage [22].

### Instrument validity and reliability

The face and content validity of the study instruments were determined by a panel of five experts from the Nursing Administration Departments affiliated with Ain Shams and Cairo Universities in Cairo, Egypt. Prior to expert review, the instruments were translated into Arabic. A separate linguist then performed a literal, word-for-word back-translation into English. The back-translated version was compared with the original to ensure accuracy and conceptual equivalence.

Then, each expert independently evaluated the relevance of each item using a 4-point Likert scale (1 = not relevant, 4 = highly relevant). The Item-Level Content Validity Index (I-CVI) was calculated for each item as the proportion of experts rating the item. Items with an I-CVI of Professional Competency Scale-Short Form (NPC-Scale SF) and The Nurses’ Moral Courage Scale (NMCS) were acceptable.

For the Professional Competency Scale-Short Form (NPC-Scale SF), internal consistency reliability was confirmed with a Cronbach’s alpha of 0.89. The Cronbach’s alpha value for each dimension including nursing care, values-based nursing care, medical and technical care, care pedagogic, documentation and administration of nursing care, and leadership development and nursing care organization ranged from 0.761 to 0.876. For dimensions, nursing care value of 0.871, values-based nursing care value of 0.761, medical and technical care value of 0.814, care pedagogic value of 0.782, documentation and administration of nursing care value of 0.876, and leadership development and nursing care organization value of 0.842 which indicating high internal consistency. Additionally, the Kaiser-Meyer-Olkin (KMO) test yield a value of 0. 941 and the validity indices for each dimension vary from 0.792 to 0.910. For dimensions, nursing care value of 0.871, values-based nursing care value of 0.761, medical and technical care value of 0.792, care pedagogic value of 0.920, documentation and administration of nursing care value of 0.897, and leadership development and nursing care organization value of 0.910 [23].

The Nurses’ Moral Courage Scale (NMCS) overall reliability was evidenced by a Cronbach’s alpha of 0.910. The Cronbach’s alpha value for each dimension including, compassion and true presence, moral responsibility, moral integrity, and commitment to providing good care ranged from 0.753 to 0.903. For dimensions, compassion and true presence value of 0.881, moral responsibility value of 0.753, moral integrity value of 0.903, and commitment to providing good care value of 0.792 which signifying high internal consistency. The Kaiser-Meyer-Olkin (KMO) test yield a value of 0. 952 and the validity indices for each dimension vary from 0.799 to 0.921. For dimensions, compassion and true presence value of 0.799, moral responsibility value of 0.913, moral integrity value of 0.921, and commitment to providing good care value of 0.853.

### Ethical considerations

We confirm that our study was conducted in accordance with the principles outlined in the Declaration of Helsinki. The study was approved by the Research Ethics Committee of the Faculty of Nursing / Ain Shams University, Cairo, Egypt (Code No. NUR 24.2.129), in accordance with the committee’s ethical standards. An official letter including the study title and objectives was sent by the Dean of the Faculty of Nursing, Ain Shams University, to the Director of Ain Shams University Hospital to obtain permission for data collection in the aforementioned setting. In addition, all study participants were informed of their right to withdraw from the study at any time and for any reason, without any consequences. Each participant provided formal written consent after receiving a full explanation of the study’s purpose and procedures. The participants were assured that all information collected would be treated confidentially and used solely for research purposes.

### Pilot study

Pilot samples were collected at the beginning of March 2024. The pilot study was conducted with eighteen staff nurses affiliated with Ain Shams University Hospital, Cairo, Egypt, representing 10% of the total study sample. The purpose of the pilot study was to assess the usability, practicality, and clarity of the study tools, as well as to estimate the time required for completion. On average, the tools took approximately 20–25 min to be completed. The data collected from the pilot study were analyzed, and the participants were subsequently included in the main study sample, as they met the inclusion criteria.

### Data collection

Data were collected over a period of six weeks, from the second half of April 2024 to the end of May 2024. The Faculty of Nursing at Ain Shams University officially approved the data collection process for this study. The researchers were present five days a week across morning, afternoon, and night shifts to ensure accessibility to all staff nurses. The research team began by introducing themselves to the nursing staff and explaining the purpose and objectives of the study. After the purpose of the study was outlined, each nurse provided written and verbal consent. Following the researchers’ explanation of how to complete the questionnaires, each nurse was given was given approximately 20–25 min to complete it.

### Statistical analysis

Data entry and statistical analysis were performed via the SPSS 20.3 statistical software package. The data are presented as frequencies and percentages for qualitative variables. Additionally, the quantitative data are presented as the means/SDs as appropriate. Internal consistency reliability was evaluated using Cronbach’s alpha coefficient, and to assess sampling adequacy and the factorability of the data for construct validity analysis, the Kaiser-Meyer-Olkin (KMO) test. Pearson’s correlation coefficient was used to assess the relationship between professional competency and moral courage.The significance level was set at *p* < 0.05 for all statistical tests and confidence intervals were set at 95%. Bootstrap methods based on 172 samples were used to ensure robustness of the findings.

## Results

### Personal and job characteristics of the study subjects

Table 1, presents that 42.7% (*n* = 73) of the staff nurses in the study were aged 25–35, 61.4% were females (*n* = 105), 64.9% were married (*n* = 111), and 28.7% had a nursing technical degree (*n* = 49). A total of 51.5% of the nurses had more than 5 years of experience (*n* = 88), 64.3% worked in non-critical care units (*n* = 110), and 57.3% did not attend training courses (*n* = 98).

### Mean score of nurses’ professional competency level

Table 2, shows that more than three-fifths of the studied subjects in the present study reported a moderate level of professional competency (61%), followed by a low level (23.3%). Inform and educate groups of patients and relatives and “interacting with other professionals in care pathways” were reported with high mean scores (3.88 ± 1.637 and 3.52 ± 2.592, respectively).

### Mean score of nurses’ moral courage (*n* = 172) level

Table 3, shows that two-thirds of the studied subjects in the present study reported a moderate level of moral courage (58.2%), followed by a low level (29%), someone else acts professionally dishonestly (e.g., steals medication from the ward), I bring it up for discussion”, and “Regardless of the care situation, I try to encounter each patient as a dignified human being, even if someone else to disagree with my doing so” were reported with high mean scores (3.88 ± 1.229, and 3.30 ± 1.205, respectively).

### Correlations between the study variables (*n* = 172)

Table 4, reveals that the professional competency of nurses was positively correlated and significantly correlated with moral courage among nurses (*r* = 0.637**, *p* = 0.000).

## Results

**Table 1:** Personal and job characteristics of the studied sample (*n* = 172)

Personal and job characteristics		Frequency	Percentage
Age	Less 25 Years	47	27.5%
	25–35 Years	73	42.7%
	More Than 35 Years	51	29.8%
Gender	Female	105	61.4%
	Male	66	38.6%
Marital Status	Married	111	64.9%
	Unmarried	60	35.1%
Educational Qualification	Nursing Diploma Degree	46	26.9%
	Nursing Technical Degree	49	28.7%
	Bachelor Nursing Degree	47	27.5%
	Master Nursing Degree	14	8.2%
	Doctorate Nursing Degree	15	8.8%
Years of Experiences	Less Than 1 Year	45	26.3%
	1–5 Years	38	22.2%
	More Than 5years	88	51.5%
Workplace Unit	Non-Critical Care Unit	110	64.3%
	Critical Care Unit	61	35.7%
Attended Training Course	Yes	73	42.7%
	No	98	57.3%

**Table 2:** Mean score of nurses̍ professional competency (*n* = 172)

Professional competency items	Mean	Std. Deviation	Level	Frequency	Percent
**Nursing care, 5 items**					
Autonomously apply the nursing process	3.10	± 1.222	Low (35–105)	40	23.3%
Meet patient’s basic physical requirements	2.90	± 0.986			
Meet patient’s specific physical requirements	3.15	± 1.001			
Document patient’s physical status	2.88	± 0.873			
Document patient’s emotional status	2.97	± 1.045			
**Value-based nursing care, 5 items**					
Respectfully communicate with patients, relatives and staff	2.95	± 1.166			
Show respect for patient independence, honesty and dignity	2.88	± 0.942			
Enhance patients’ and relatives’ knowledge and experiences	2.85	± 0.974			
Show admiration for different values and beliefs	2.95	± 1.036			
Contribute to a complete view of the patient	3.01	± 1.177			
**Medical and technical care**,** 6 items**					
Manage drugs and clinical request of knowledge in pharmacology	2.84	± 1.167			
Independently administer prescriptions	2.83	± 1.172			
Pose questions about blurred instructions	2.90	± 1.429			
Support patients during examinations and treatments	2.83	± 1.311			
Follow up on patient’s conditions after examinations and treatments	3.10	± 1.564			
Handle medical/technical equipment according to regulation and safety routines	3.05	± 1.578			
**Care pedagogics**,** 5 items**					
Provide patients and relatives with provision to enhance sharing in patient care	3.02	± 1.406	Moderate (106–131)	105	61%
Inform and educate individual patients and relatives	2.94	± 1.431			
Inform and educate groups of patients and relatives	3.88	± 1.637			
Make sure that information given to the patient is understood	2.90	± 1.644			
Inspire the patient to follow treatments	2.85	± 1.683			
**Documentation and administration of nursing care**,** 8**					
Make use of applicable data in patient records	2.94	1.771	High (132–175)	27	15.7%
Use information technology as a provision in nursing care	3.28	± 1.836			
Document according to current legislation	2.98	± 1.818			
Comply with current legislation and routines	3.03	± 1.969			
Switch sensitive personal data in a safe way	3.00	± 1.988			
Detect work-related risks and prevent them	3.18	± 2.140			
Continuously engage in professional development	3.03	± 2.090			
Lead and develop health staff teams	3.05	± 2.253			
**Development**,** leadership and organization of nursing care**,** 6 items**					
Act sufficiently in the event of unethical conduct among employees	2.97	± 2.273			
Apply philosophies of disaster medicine	3.22	± 2.357			
Search and appraisal relevant literature for evidence-based nursing	2.74	± 2.493			
Cooperate with other professionals in care pathways	3.52	± 2.592			
Teach, supervise and measure students	3.00	± 2.471			
Oversee and educate staff	3.44	± 2.713			

**Table 3:** Mean score of nurses’ moral courage (*n* = 172)

Moral courage items	Mean	Std. Deviation	Level	Frequency	Percent
**Compassion and true presence (5 items)**					
1-I support a suffering patient by being truly present for him/her, even if it were to lead me to meeting my own inner fears	2.83	± 0.701	Low (21–63)	50	29%
2-Regardless of the care state, I try to encounter each patient as a dignified human being, even if someone else were to distress with my doing so	3.30	± 1.205			
3-Regardless of the care state, I seek to create a genuine human encounter with the patient, even though a more superficial relationship would be easier for me	3.10	± 1.133			
4-In order to ensure moral care for my patient I do not avoid even difficult	2.91	± 0.942			
5-I discuss the fears caused by the illness with my patient even if it would lead me to face my own inner fears.	2.79	± 1.004			
**Moral responsibility (4 items)**					
6-I participate in the care team’s ethical decision-making regardless of somebody else	3.13	± 1.287			
7-I contribute in the care team’s ethical decision making ethical problem situations often involve doubt as to the right answer	3.01	± 1.233			
8-I bring up for discussion the patient’s right to good care if someone else* insists that I compromise on loyalty to the ethical principles of health care	3.05	± 1.008			
9-I participate in care team’s ethical decision-making ethical problematic situations often	2.91	± 0.960			
**Moral integrity (7 items)**					
10-If someone else acts professionally unfairly (e.g., steals medication from the ward), I bring it up for discussion	3.88	± 1.229	Moderate (64–78) (106–131)	100	58.2%
11-If someone else tries to cover up an evident care error he/she has made, I bring it up for discussion	2.86	± 1.181			
12-I adhere to professional ethical principles even if I were to be bullied for it in my work unit	2.88	± 1.205			
13-I bring up for conversation an ethical problem situation that arises in nursing care even if someone else wants to remain silent about it	2.89	± 1.249			
14-If someone else acts unethically, I bring it up for discussion, even if I were to get negative feedback for it in my public work	3.24	± 1.314			
15-I acknowledge my own mistakes in care (e.g., administering the wrong medication to a patient)	2.88	± 1.290			
16-I act in accordance with professional ethical values even if someone else.	2.93	± 1.417			
**Commitment to good care (5 items)**					
17-I am even prepared to break predominant care practices to advocate my patient (e.g., to exceed the standard length of time prescribed for a care procedure if it is inadequate for good care)	2.99	± 1.368	High (79–105)	22	12.8
18-If the resources required for ensuring good care are insufficient (e.g., inadequate staff) I bring it up for discussion	3.12	± 1.583			
19-If I observe evident deficiencies in someone else’s professional competence, I bring it up for discussion	2.99	± 1.481			
20-someone else compromises on adherence to the ethical principles of health care (human self-respect, independence, and justice)	3.02	± 1.663			
21- I do not compromise on my patient’s right to good care even though someone else were to bully me into doing so	3.09	± 1.781			

**Table 4:** Correlations between the study variables (*n* = 172)

Variable		Moral courage
**Professional competency**	Pearson Correlation	0.637**
	Sig. (2-tailed)	0.000

**. Correlation is significant at the 0.01 level (2-tailed)

Unless otherwise noted, bootstrap results are based on 172 bootstrap samples

## Discussion

This study aimed to assess staff nurses’ self-reported professional competency level, measuring staff nurses’ moral courage level, and finding out the relationship between professional competency and moral courage among staff nurses.

### Professional competency

With respect to professional competency, the present study findings revealed that more than three-fifths of the studied subjects had a moderate level of professional competency, followed by less than one-quarter of the studied subjects, who had a low level. Similarly, a study conducted by [1–4, 26–27] further examined nurses’ professional competency and reported moderately level which supports the present findings. In contrast, two studies were conducted in Iran reported that most nurses rate their overall level of professional competence as very good level/ high [28–29].

A moderate level of professional competency could be explained as nurses possess adequate basic skills and knowledge for daily nursing tasks. However, they may struggle with complex clinical decision-making, leadership responsibilities, or applying advanced evidence-based practices. This may affect the consistency and safety of care, especially in high-acuity or emergency situations. This could be due to factors such as limited exposure to continuing education, restricted opportunities for advanced clinical training, or insufficient practical experience in complex patient care scenarios [27–31].

This level often necessitates additional support, supervision, and ongoing education to ensure safe and effective practice. Conversely, a low level of professional competency suggests critical gaps in fundamental nursing responsibilities, which may lead to increased clinical errors, ineffective communication, and reduced patient satisfaction [31, 32]. Nurses in this category require close supervision and targeted skill-building programs. This could be due to poor educational background, lack of institutional support for professional development, high workload, and low motivation or job dissatisfaction [30, 31]. Such deficiencies underscore the urgent need for targeted training programs and mentorship to elevate competency levels.

In this regard a study by [5] found a notable correlation emerged in their study between the number of workshops attended by nurses and their competence levels across all competency domains. In addition, a recent study in Japan showed that attending a two-day international outreach seminar provided participants with valuable and current knowledge regarding the competency of nurse educators. Also, the same study highlighted that “Care Pedagogics” domain underscores the crucial role of nurses in educating and supporting patients and their families. These results emphasize the ongoing need to prioritize clinical proficiency in nursing education and practice [22]. Additionally, Egyptian studies concluded that workshops had a beneficial impact on enhancing the knowledge, collaboration skills, and overall performance of both head and staff nurses [33].

Furthermore, it’s critical to stress how important it is for nurses to maintain quality of life. To guarantee that high-quality care is provided, initiatives to enhance the quality of life for nurses must be initiated. When creating programs to improve nurses’ competence, nurse managers should take the results into account [34] and use reflective learning, which can help nurses to develop a good self-perception of their competence [35].

From the researchers’ perspective, the variations observed between the findings of this study and those previously mentioned could be attributed to differences in the demographics of the samples and the specific instruments used to evaluate professional competency. Importantly, the level of professional competency in nursing is crucial and shaped by elements such as the design of the education system, perceptions of nursing, socioeconomic conditions, and cultural traits. Issues such as low motivation, high burnout, poor quality of education, dissatisfaction with jobs, and irregular hiring practices in relation to patient care, along with the lack of defined standards for professional competency, can lead to a decrease in the clinical competencies of nurses. Studies that focus on qualitative data have noted that aspects such as experience, opportunities for advancement, the work setting, personal traits, motivation, and theoretical knowledge play a significant role in determining the clinical competencies of nurses.

### Moral courage

Regarding moral courage, more than half of the nurses who participated in this study had a moderate level of moral courage, followed by more than one quarter who had a low level of moral courage. This finding was the same as that of a study conducted by [36–37], who reported that their subjects reported a moderate level of moral courage. Nonetheless, the results of a study conducted by [9, 38–41] revealed that nurses enjoy a high level of moral courage. The diverse results of different studies may be attributed to disparities in the work environment, ethical atmosphere, organizational culture, organizational and managerial support, fear of social seclusion, collective thought, and lack of acceptance by the organization [9].

From the researchers’ perspective, this could be attributed to the variations in the timing of each study and the unique circumstances of the research setting, including the organizational frameworks and the cultural and ethical values of the community being studied. Also, the study suggests that although nurses are ethically aware and willing to act correctly in routine care situations, they may hesitate in the face of ethical dilemmas or institutional pressure. This hesitation may compromise ethical advocacy and patient safety, particularly in high-stakes settings [42]. Cultural factors in Egypt such as a collectivist orientation, respect for authority, and a tendency to avoid conflict may also contribute to this outcome. These values, while promoting social cohesion, can inhibit assertiveness and moral action in professional settings [45, 46]. To foster moral courage, it is essential to provide structured ethics education, institutional support, and leadership models that empower nurses to speak up and act with integrity despite potential risks [42, 43].

Nurses with high moral courage are more likely to report unsafe practices, protect patients’ rights, and challenge unethical decisions, even when facing personal or professional risks. Without such courage, ethical silence may occur, potentially compromising patient well-being and the integrity of nursing care [42, 44]. Moreover, the organizational and ethical climate of the workplace significantly influences moral courage. Work environments that promote ethical leadership, clear communication, and justice are associated with higher levels of moral courage. Conversely, unsupportive or punitive environments may discourage nurses from acting according to their ethical beliefs [45–46].

### Correlations between the study variables

The present study revealed that the professional competency of nurses was positively and significantly correlated with moral courage among nurses. This may be attributed to the ethical and moral competence components of professional competence to enact a nurse’s profession. This finding came is in agreement with [8], who reported that professional competence directly and positively affects moral courage. As such, nurses need to learn and develop virtue ethics and principles to enact acceptable levels of moral courage and become morally courageous in their professional journeys. Similarly [47] emphasized that morally courageous nurses tend to possess higher professional standards and are more committed to patient advocacy and ethical decision-making. These findings support the idea that moral courage is not only an ethical trait but also an expression of professional mastery and confidence.

However, not all studies have confirmed this direct correlation. For instance [48], found that even highly competent nurses may remain silent in ethically charged situations due to workplace culture, hierarchical pressure, or fear of retaliation, indicating that external barriers can suppress moral action regardless of a nurse’s clinical capabilities. Likewise [44], noted that moral distress and institutional constraints may override personal competencies, suggesting that competency alone may not guarantee morally courageous behavior. These contrasting perspectives highlight that while competency is a critical enabler of moral courage, it must be supported by a healthy ethical climate, supportive leadership, and a culture that encourages speaking up. Therefore, in addition to strengthening nurses’ skills, healthcare organizations must also prioritize ethical empowerment and psychological safety to fully translate competency into courageous moral action.

From the researchers’ point of view, the positive correlation between professional competency and moral courage reflects a structural interdependence between applied knowledge and ethical behavior in nursing practice. Nurses with a higher level of professional competence are more aware of best practices and more capable of distinguishing between ethically sound and unsound actions. This awareness fosters self-confidence, which is a crucial driver of morally courageous behavior when facing ethical dilemmas or value-based conflicts [37]. Furthermore, the researchers suggest that moral courage is not acquired in isolation from clinical expertise. Rather, the educational environment and real-world experiences encountered during professional development play a significant role in shaping a nurse’s moral reasoning [46]. As clinical experience and competence increase, so does the nurse’s ability to defend ethical decisions especially in environments that may lack managerial support or have unclear boundaries of responsibility.

However, this relationship is not automatic or guaranteed. It is also influenced by organizational and contextual factors such as leadership style, the clarity of institutional values, opportunities for ethical dialogue, and the extent to which nurses’ voices are respected within the team. prior studies have noted that even highly competent nurses may remain silent in ethically complex situations when faced with workplace intimidation, fear of retaliation, or ethical distress [44–48]. Therefore, while enhancing clinical competence is essential, it must be accompanied by an ethically supportive environment that enables nurses to exercise moral courage without fear of negative consequences. Consequently, this helps them uphold their identity as healthcare professionals, efficiently and compassionately fulfill their duties and responsibilities, and protect their emotional and psychological health.

## Conclusion

This study concluded that more than three-fifths of the studied subjects in the present study reported a moderate level of professional competency (followed by a low level), and two-thirds of the studied subjects in the present study reported a moderate level of moral courage, followed by a low level. Finally, the professional competency of nurses was positively correlated and significantly correlated with moral courage among nurses (*r* = 0.637**, *p* = 0.000).

### Implications for practice and future research

Training and educational programs should be held continuously and periodically to update and expand nurses’ knowledge and skills concerning professional competencies and the implementation of ideal models in clinical practice. Additionally, regularly assessing nurses’ knowledge to identify topics and areas that need to be covered in training courses is essential for improving their professional competencies. Moreover, creating programs aimed at improving the professional competencies of nurse supervisors and their ability to support staff nurses effectively is crucial. To strengthen the foundation of professional competencies, it is equally important to integrate these training priorities into undergraduate and postgraduate nursing education, ensuring that future nurses are well-prepared for clinical demands. Eventually, conducting a study on the impact of nurses’ professional competencies on their empowerment would provide valuable evidence to guide both educational and clinical practices.

### Limitations of the study

First, one of the limitations of this study was that, it was based on participants’ self-reported data which reliance on self-reported responses in the tools which may introduce response bias or inaccuracies, affecting reliability of the results. All the participants gained anonymity and confidentiality, but they could still not completely avoid reaction bias. The study involves only one hospital which may limit the ability to generalize the study results.

## Data Availability

Owing to confidentiality concerns, the data and materials used in the current research cannot be made publicly available. However, they are available from the corresponding author upon reasonable request.
